# Life experiences associated with change in perpetration of domestic violence

**DOI:** 10.1186/s40621-020-00264-z

**Published:** 2020-08-01

**Authors:** Avanti Adhia, Vivian H. Lyons, Hannah Cohen-Cline, Ali Rowhani-Rahbar

**Affiliations:** 1 Firearm Injury & Policy Research Program, Harborview Injury Prevention & Research Center, University of Washington, Box 359960, 325 Ninth Ave, Seattle, WA 98104 USA; 2 Department of Epidemiology, School of Public Health, University of Washington, 1959 NE Pacific Street, Health Sciences Building, Box 357236, Seattle, WA 98195 USA; 3 Center for Outcomes Research and Education, Providence Health & Services, 5251 NE Glisan Street, Portland, OR 97213 USA

**Keywords:** Domestic violence, Intimate partner violence, Perpetration, Substance use

## Abstract

**Background:**

This study assessed whether several adult life experiences, including loss of support, loss of food security, loss of housing, and substance use cessation, are associated with change in domestic violence (DV) perpetration from early to later adulthood. Using 2015 to 2016 cross-sectional, self-report survey data from Medicaid enrollees in Oregon (*N* = 1620), we assessed change in DV perpetration from early adulthood (19–30 years) to later adulthood (≥ 31 years of age), cut points determined by existing survey questions. Multinomial logistic regression models were constructed to estimate the association between life experiences and physical DV perpetration using odds ratios (OR), adjusting for sociodemographic characteristics, DV victimization, and childhood abuse, bullying, and social support.

**Findings:**

Of the 20% of participants who perpetrated DV, 36% perpetrated DV in both early and later adulthood (persisters), 42% discontinued (desisters) and 22% began (late-onsetters) perpetration in later adulthood. Loss of support and loss of food security were both associated with change in DV perpetration (i.e., desistance or late onset of perpetration or both). Loss of support was associated with 9.5 times higher odds of being a desister (OR = 9.5, 95% CI = 1.1, 84.1) and 54.2 times higher odds of being a late-onsetter (OR = 54.2, 95% CI = 6.5, 450.8) of DV perpetration compared to persisters. Loss of food security was associated with 10.3 times higher odds of being a late-onsetter (OR = 10.3, 95% CI = 1.9, 55.4) of DV perpetration compared to persisters. In addition, substance use cessation was associated with 10.3 times higher odds of being a desister (OR = 10.3, 95% CI = 1.9, 56.2) compared to persisters.

**Conclusions:**

Findings suggest that specific life experiences in adulthood, including loss of support, loss of food security, and substance use cessation, are associated with changes in DV perpetration.

## Introduction

Domestic violence (DV) is a substantial public health problem with severe and long-lasting consequences and considerable economic burden for society (Peterson et al. 2018). DV includes physical violence, sexual violence, stalking and psychological harm and can include violence perpetrated by intimate partners, immediate family members, or other relatives (Garcia-Moreno et al. 2006; Smith et al. 2018). The majority of DV is perpetrated by intimate partners (i.e., current or former partners or spouses) (Truman and Morgan 2015), and DV is a term often used to refer interchangeably to intimate partner violence (IPV) (Costa et al. 2015). In this study, we use DV to refer to violence against partners and loved ones including other family members, while IPV refers to violence specifically between intimate partners. Although the focus of this study is on DV, prior research has largely focused on IPV given that it constitutes the majority of DV, so we cite IPV literature throughout this article and use the terminology as defined above.

Nationally representative data of adults in the US indicate that 36.4% of women and 33.6% of men report experiencing sexual violence, physical violence, and stalking by an intimate partner in their lifetime (Smith et al. 2018). Challenges of gathering accurate estimates of perpetration through surveys have led to less data on IPV perpetration than victimization with the majority of studies focused on male perpetration in a heterosexual relationship (Fulu et al. 2013), but a meta-analysis of perpetration rates estimated that approximately a quarter of individuals reported ever perpetrating physical violence against a partner (Desmarais et al. 2012).

Prior literature has identified several determinants of DV perpetration. Childhood factors including history of abuse and witnessing IPV are associated with perpetrating IPV later in life (Capaldi et al. 2012; Costa et al. 2015). More proximal factors have also been found to be associated with IPV perpetration, including alcohol and drug use (Capaldi et al. 2012) and other career or life stressors in adulthood like employment instability and financial strain (Stith et al. 2004). In a large national study, past-year stressors like financial crisis, a serious problem with a neighbor/friend, death of a loved one, and being fired/laid off from job were associated with IPV perpetration (Roberts et al. 2011).

While much of the prior research has focused on DV perpetration at a single point in time, fewer studies provide information about patterns of persistence (continuing), desistance (stopping), and onset (starting) of perpetration (Kim et al. 2008). Individuals can and do desist from IPV over time (Shortt et al. 2012), but there is little known about the mechanisms involved in the change process, particularly for adults (Giordano et al. 2015). Furthermore, little is known about differences between individuals who begin or discontinue IPV perpetration and those who perpetrate IPV throughout adulthood (Walker et al. 2013). Identifying factors that lead individuals to change, and particularly cease, their use of IPV can shed light on interventions that may be effective in initiating desistance (Walker 2017; Walker et al. 2015). This is especially important as batterer intervention programs remain only marginally effective (5% reduction) at reducing IPV perpetration (Babcock et al. 2004).

In this study, we assessed the association between several adult life experiences, including the loss of support, loss of food security, loss of housing, and substance use cessation, and change in physical DV perpetration from early to later adulthood. We hypothesized that each of these four adult life experiences would be associated with a change (i.e., desistance or onset) in physical DV perpetration from early to later adulthood. The selection of these four life experiences are especially compelling as they have been identified as proximal factors for IPV perpetration (Capaldi et al. 2012; Roberts et al. 2011; Stith et al. 2004) and are concrete, potentially modifiable life experiences which could be incorporated into batterer treatment programs to increase their efficacy. To our knowledge, no prior study has examined these life experiences in the context of persisting, desisting or late onset of physical DV perpetration.

## Methods

### Participants

We used data from the LIFE Experiences Study, a cross-sectional survey designed to explore the connections between life experiences and health outcomes of adult Medicaid enrollees in the Portland, Oregon metro area (Cohen-Cline et al. 2019). Eligible individuals were between 18 and 65 years of age and enrolled in Medicaid for at least 6 months during the prior year with a valid mailing address. A representative sample of over 9000 individuals was selected for the survey. From 2015 to 2016, 2386 individuals returned completed surveys (response rate = 26%). Participants were excluded if their age was unknown (*n* = 21, 1%) or < 31 years (*n* = 225, 9%) since our analysis aimed to assess changes in experiences between “early” (ages 19–30) and “later” (age 31+) adulthood. We also excluded 4 participants (< 1%) with unknown survey weights. We used multiple imputation to account for missingness in DV perpetration (*n* = 251, 12% missing), the four life experience predictors (*n* = 386, 18% missing), and covariates (*n* = 201, 9%). The final analytic sample included 2136 individuals aged ≥31 years. The periods of adulthood, “early” (ages 19–30) and “later” (age 31+), were pre-defined in the survey questions (see Additional file 1 for further sample details and exact survey questions). The Providence Health & Services Institutional Review Board approved this study.

### Measures

#### Primary exposures

All exposures were assessed separately for early adulthood (ages 19–30) and later adulthood (age 31+). We focus on four adult life experiences, including three risk factors (loss of support, loss of food security, loss of housing) and one protective factor (substance use cessation). For each item, participants marked yes or no for early adulthood and yes or no for later adulthood. Participants were asked if they had close relationships with people they could count on and were considered to have loss of support if they responded yes in early adulthood and no in later adulthood. Food security was operationalized as having trouble affording enough to eat. If participants responded no in early adulthood and yes in later adulthood, they were considered to have loss of food security. Loss of housing was marked as a change from never homeless in early adulthood to ever homeless in later adulthood. Participants were considered to have used substances if they answered yes to the question on being a problem drinker, alcoholic or user of street drugs. Substance use cessation was identified if participants responded yes in early adulthood and no in later adulthood.

#### Primary outcome

Physical DV perpetration was assessed with the question “Did you slap, hit, kick, punch, or beat up a partner or loved one?” separately for early and later adulthood. Response options were yes or no to indicate any perpetration within each time period. Participants were categorized into four groups based on persistence of DV from early adulthood to later adulthood. “Persisters” perpetrated DV in both early and later adulthood, “desisters” discontinued DV perpetration in later adulthood, “late-onsetters” began DV perpetration in later adulthood, and “never perpetrators” did not perpetrate DV in either early or later adulthood.

#### Covariates

Covariates were selected based on their established associations with adult DV perpetration from prior literature (Capaldi et al. 2012; Costa et al. 2015). Sociodemographic characteristics included sex (male or female), age (continuous in years), race/ethnicity (White, Black, Hispanic, and all other races), and gross past-year household income (coded categorically: ≤$5000, $5001–$10,000, $10,001–$20,000, and > $20,000 given small sample sizes for higher income categories in Medicaid population). Three childhood variables through age 18 years were included: 1) child abuse by an adult was assessed with three questions on emotional/psychological, physical and sexual abuse and coded as any vs. none; 2) bullying by a peer or classmate was coded as yes or no; and (3) childhood social support was assessed with the question, “Did you generally have close, supportive relationships with family and/or friends?” and coded as yes or no. In addition, physical DV victimization as an adult (in either early or later adulthood) was assessed with the question “Were you slapped, hit, kicked, punched, or beat up by a partner or loved one?” and coded as yes or no.

### Analysis

We first examined prevalence and patterns of DV perpetration from early adulthood to later adulthood. For all primary predictors and covariates, we examined prevalence overall and by DV perpetration category. Chi-squared (χ^2^) tests were used to test for significant differences across DV perpetration groups. Among individuals who reported perpetrating DV, multinomial logistic regression was used to assess the association between adult life experiences (i.e., loss of support, loss of food security, loss of housing, and substance use cessation) and pattern of physical DV perpetration (i.e., persisters, desisters, late-onsetters), adjusting for sex, age, race/ethnicity, income, childhood abuse, childhood bullying, childhood support, and physical DV victimization. Persisters served as the reference group. Coefficients were exponentiated to obtain odds ratios.

To account for missing data on the variables of interest (i.e., DV perpetration, adult life experiences, and covariates), multiple imputation methods using chained equations were implemented using the “mi” suite of commands in Stata to pool results from 20 imputed data sets. To check the sensitivity of our findings, we compared the regression results with multiply imputed data to the results with complete data (*n* = 1617) and found no substantive differences. Survey weights were used in all analyses so that results reflect Medicaid enrollees in Portland. The Additional file 1 contains detailed information about sampling, survey weighting, and exact survey question wording. All analyses were conducted using Stata 15.1.

## Results

Table 1 presents observed data of the analytic sample. The sample was 39.4% male and 67.5% white. Participants were ages 31–77 years (mean = 51 years), and 34.2% had a past-year household income of ≤$5000. Overall 79.7% of participants never perpetrated DV, while 7.3% were persisters, 8.5% were desisters, and 4.5% were late-onsetters. Among only those reporting perpetrating DV in either period of adulthood, 35.9% were persisters, 41.7% were desisters, and 22.3% were late-onsetters. Overall, 6.9% of the sample reported loss of support, 9.8% reported loss of food security, 15.2% reported loss of housing, and 13.5% reported substance use cessation from early to later adulthood (Table 1). χ^2^-tests showed significant differences across perpetration groups for loss of support and substance use cessation, with late-onsetters reporting the highest proportion of loss of support (27.0%), and desisters reporting the highest proportion of substance use cessation (41.2%). Compared to persisters and late-onsetters, desisters and never perpetrators had a higher proportion of white individuals. Overall, 55.2% of the sample had a history of childhood abuse and 35.4% experienced physical DV. Never perpetrators were less likely to have a history of child abuse and were less likely to have experienced physical DV compared to all other perpetration groups. There were no significant differences in sex, age, income, childhood bullying, and childhood support across perpetration groups.

**Table 1:** Distribution of observed participant characteristics by domestic violence perpetration category in the LIFE Experiences Study

	Total (*n* = 1617)	Persisters (*n* = 154)	Desisters (*n* = 128)	Late-Onsetters (*n* = 87)	Never Perpetrators (*n* = 1248)	*p*-value^a^
		% (n)				
**Primary predictors**						
*Risk factors*						
Loss of support	6.9 (107)	1.9 (11)	12.7 (9)	27.0 (13)	5.6 (74)	0.04
Loss of food security	9.8 (184)	1.8 (12)	7.6 (15)	19.4 (21)	10.2 (136)	0.06
Loss of housing	15.2 (268)	26.4 (25)	23.5 (27)	26.1 (30)	12.7 (186)	0.20
*Protective factor*						
Substance use cessation	13.5 (189)	5.2 (7)	41.2 (38)	1.5 (5)	12.0 (139)	< 0.001
**Covariates**						
Male	39.4 (643)	32.1 (52)	49.6 (49)	31.4 (33)	39.5 (509)	0.53
Age^b^						
31–40	16.6 (194)	17.7 (13)	24.2 (22)	16.2 (5)	15.7 (154)	0.18
41–50	32.8 (375)	19.7 (39)	49.1 (38)	14.3 (15)	33.3 (283)	
51–60	28.9 (516)	39.3 (53)	13.3 (33)	48.8 (43)	28.5 (387)	
61+	21.7 (532)	23.4 (49)	13.4 (35)	20.7 (24)	22.5 (424)	
Race/ethnicity						
White	67.5 (1076)	39.5 (75)	66.1 (75)	49.1 (57)	71.3 (869)	0.003
Black	8.2 (190)	18.6 (34)	3.4 (15)	15.3 (11)	7.3 (130)	
Hispanic	7.0 (111)	2.6 (12)	20.2 (18)	7.6 (6)	5.9 (75)	
All other	17.4 (240)	39.4 (33)	10.3 (20)	28.1 (13)	15.5 (174)	
Income						
≤ $5000	34.2 (579)	45.1 (71)	41.8 (41)	44.8 (34)	31.9 (433)	0.71
> $5000 – $10,000	18.5 (379)	17.2 (35)	22.2 (33)	16.3 (21)	18.3 (290)	
> $10,000 – $20,000	18.5 (358)	15.2 (28)	8.5 (31)	11.5 (19)	20.2 (280)	
> $20,000	28.9 (301)	22.5 (20)	27.6 (23)	27.4 (13)	29.7 (245)	
Childhood abuse	55.2 (1015)	91.5 (129)	69.8 (102)	85.0 (70)	48.7 (714)	< 0.001
Childhood bullying	53.2 (914)	73.6 (93)	65.5 (91)	53.2 (54)	50.0 (676)	0.10
Childhood support	68.0 (1061)	58.9 (85)	61.8 (76)	55.0 (49)	70.2 (851)	0.41
Physical DV victimization	35.4 (682)	64.2 (113)	53.5 (82)	69.4 (59)	28.9 (428)	< 0.001

Note: Percentages shown are survey weighted. Sample sizes are unweighted, and variables are presented with no imputation for missingness. Missing data: DV perpetration (*n* = 251, 12%), loss of support (*n* = 240, 11%), loss of food security (*n* = 260, 12%), loss of housing (*n* = 104, 5%), substance use cessation (*n* = 248, 12%), covariates (*n* = 201, 9%)

^a^*p* values based χ^2^-tests

^b^Age is included as a continuous variable in the analysis but is shown descriptively in categories in this table

Table 2 presents the multinomial logistic regression coefficients. Loss of support was associated with 9.5 times higher odds of being a desister (aOR = 9.5, 95% CI = 1.1, 84.1) and 54.2 times higher odds of being a late-onsetter (aOR = 54.2, 95% CI = 6.5, 450.8) of DV perpetration compared to persisters. That is, the likelihood of desistance and late-onsetting was higher for individuals experiencing loss of support. Loss of food security was associated with 10.3 times higher odds of being a late-onsetter (aOR = 10.3, 95% CI = 1.9, 55.4) and was not associated with significantly higher odds of being a desister compared to persister. Loss of housing was not associated with significantly higher odds of being a desister or late-onsetter of DV perpetration compared to persisters. Substance use cessation was associated with 10.3 times higher odds of being a desister (aOR = 10.3, 95% CI = 1.9, 56.2) compared to persisters. Substance use cessation was not associated with significantly higher odds of being a late-onsetter compared to persisters.

**Table 2:** Change in life experiences and perpetration group among perpetrators of physical domestic violence

	Unadjusted	Adjusted^a^
	Odds Ratio (95% Confidence Interval)	
*Risk factors*		
Loss of support		
Persisters	1.00	1.00
Desisters	6.18 (0.69, 55.13)	**9.46 (1.06, 84.11)**
Late-onsetters	**12.89 (2.17, 76.51)**	**54.19 (6.51, 450.84)**
Loss of food security		
Persisters	1.00	1.00
Desisters	2.57 (0.68, 9.65)	1.68 (0.34, 8.37)
Late-onsetters	**7.00 (1.66, 29.53)**	**10.33 (1.93, 55.41)**
Loss of housing		
Persisters	1.00	1.00
Desisters	0.83 (0.16, 4.17)	0.91 (0.28, 3.00)
Late-onsetters	1.83 (0.35, 9.46)	2.01 (0.49, 8.28)
*Protective factor*		
Substance use cessation		
Persisters	1.00	1.00
Desisters	**11.12 (2.59, 47.73)**	**10.31 (1.89, 56.16)**
Late-onsetters	0.29 (0.06, 1.31)	0.26 (0.03, 1.89)

Note: Multiple imputation was implemented to address missingness for all variables of interest (i.e., DV perpetration, life experiences, and covariates)

Boldface indicates statistical significance (*p* < 0.05)

^a^Models adjusted for sex, age, race/ethnicity, income, childhood abuse, childhood bullying, childhood support, and physical DV victimization

## Discussion

The findings from this study highlight specific life experiences in adulthood that are associated with changes in DV perpetration. Loss of support and loss of food security were both associated with a change in DV perpetration (i.e., desistance or late onset of perpetration or both). These results highlight how changes in situational circumstances can affect the use of violence, which may have important implications for prevention of DV since background risk factors (i.e., childhood history variables, sociodemographic characteristics) may be more challenging to intervene on (Costa et al. 2015).

Prior literature has shown that particular acute (e.g., past-year) stressors in adulthood, including financial crisis or instability, problems with a neighbor or friend, death of a loved one, are associated with risk of IPV perpetration (Roberts et al. 2011; Schwab-Reese et al. 2016). Due to the cross-sectional nature of this data, we were unable to understand the temporal ordering between life experiences and changes in DV perpetration. Thus, our findings could have multiple interpretations. Social support has been shown to be related to reduced risk of IPV, and loss of such support might indicate the end of a relationship preventing the opportunity for continued perpetration (for desisters) or act as a stressor that increases risk of IPV perpetration (for late-onsetters) (Wright 2015). Similarly, loss of food security may be conceived of as a loss of financial stability, a stressor that may contribute to the onset of IPV perpetration (Capaldi et al. 2012; Schwab-Reese et al. 2016). Addressing root causes of violence perpetration, including financial instability and lack of support, may be necessary to effectively prevent DV.

Additionally, we found that substance use cessation was associated with desistance of DV perpetration. Although there is substantial evidence of an association between substance use and IPV perpetration, limited research exists specifically on the role of substances in IPV or DV cessation (Cafferky et al. 2018; Stith et al. 2004). Our findings align with prior studies showing that changing attitudes towards alcohol consumption and ending alcohol and drug use were important to the process of IPV desistance and thus might indicate a useful target for intervention (Merchant and Whiting 2018; Walker 2017). Indeed, treatment for problematic substance use has been shown to reduce IPV (Murphy and Ting 2010; Stuart et al. 2009). Although this treatment is not likely to be fully adequate for stopping DV, given that individuals in this sample who report ceasing substance use between early and later adulthood are more likely to desist from DV, DV interventions that integrate substance use treatment may be more effective in reducing use of violence for some perpetrators.

Approximately 20% of the individuals in this sample reported perpetration of DV in either period of adulthood, which is in line with national estimates (Kessler et al. 2001; Singh et al. 2014). Of those who perpetrated DV, 36% were persisters, 42% were desisters, and 22% were late-onsetters. This generally aligns with prior research that underscores that both persistence and desistance of IPV are likely within a given sample (Capaldi and Kim 2007; Walker et al. 2013). Previous studies have also found the large majority of perpetrators do desist, confirmed by our study finding that the largest group of perpetrators were desisters (Whitaker et al. 2010).

These findings must be considered in light of some limitations. DV perpetration was only assessed with a single question about physical abuse towards a partner or loved one. Thus, many types of violent behavior (e.g., sexual or psychological violence) were not included, the relationship between the perpetrator and victim (e.g., intimate partner, other family member) was not specified, and severity, motivation, and context of the physical abuse were not measured. The survey also did not assess relationship status or ask which relationships included violence, which is important since violence is not necessarily stable across relationships (Whitaker et al. 2010) and changes in perpetration may simply reflect relationship status (e.g., desisting because the relationship ended). Future research should explore patterns of and changes in DV perpetration across and within relationships. Although this study was able to examine change in perpetration status over time, only two large time periods were asked about (early vs. later adulthood), so we are likely underestimating the amount of change and do not know exact timing of life experiences. The study relied on retrospective reporting of data, which may be susceptible to recall and social desirability bias. Participants may be reporting on events that occurred recently or several decades prior depending on the question and their age at the time of the survey. The sample size of DV perpetrators was also relatively small (particularly when stratified by life experiences and pattern of perpetration), resulting in wide confidence intervals. While the exact point estimates may be imprecise, we were able to control for several important covariates and take these results as evidence that these life experiences deserve further study. Finally, the response rate to the LIFE Experiences Study was low (26%), and individuals who responded were more likely to be female, non-Hispanic White, and older than non-respondents, so results may not be generalizable to all Medicaid enrollees in Portland, Oregon.

Understanding the impact of life course experiences – including loss of support, loss of food security, and substance use cessation – on changes in DV perpetration offers potential targets for intervention. Integrating components addressing peer and family supports, food security and financial stability, and substance use into existing DV intervention programs may enable them to be more effective in reducing violence.

## Supplementary information

**Additional file 1.** Sampling, Weighting, & Survey Questions from LIFE Experiences Study.

## Data Availability

Deidentified data are available upon request and with permission of the Providence Health & Services Institutional Review Board. Requests for data can be made to Hannah Cohen-Cline (Hannah.Cohen-Cline@providence.org).
