# Supplementary material for: Life experiences associated with change in perpetration of domestic violence
Source: Inj Epidemiol. 2020 Aug 1;7:37. doi: 10.1186/s40621-020-00264-z (PMC7395385; doi:10.1186/s40621-020-00264-z)
Supplement: Supplementary file 1 — Additional file 1. Sampling, Weighting, & Survey Questions from LIFE Experiences Study. [file 40621_2020_264_MOESM1_ESM.docx]

**APPENDIX:**

**Sampling, Weighting, & Survey Questions from LIFE Experiences Study**

**SAMPLING**

The study population was drawn from a Medicaid Coordinated Care Organization (CCO) in the Portland, Oregon region. At the time of sampling, there were 134,088 adult members (ages 18-65 years), 106,146 of whom who met our inclusion criteria. Children under 18 were not included in the sampling universe.

The sampling frame included adult (18-65) Medicaid members with valid mailing addresses who had been enrolled for at least 6 months of the year prior to the study. This minimum enrollment criteria was included because at least 6 months of retrospective claims data was needed to classify patients into the sampling strata specified below.

The intent of the original study was to compare the prevalence of different adversities between members with differing health profiles, focusing on “complex high cost patients.” Thus, a stratified sampling plan was used such that all members of the sampling universe were placed into one of four mutually exclusive strata based on medical complexity (high vs. non-high) and health care utilization (high vs. non-high). Strata were defined based on claims history in the year prior to the sample draw. High medical complexity was defined as the top 10th percentile of Chronic Illness and Disability Payment System scores, a validated claims-based algorithm that scores members based on chronic medical conditions, demographics, and prescription patterns (Gilmer, Kronick, Fishman, & Ganiats, 2001). High utilization of health care was defined as three or more emergency room visits, two or more inpatient visits, or two emergency department visits and one inpatient visit in the last 12 months.

After exclusions, the final sampling universe consisted of 106,146 adults distributed across our four primary sampling strata. Sampling targets were set by these strata and three race/ethnicity substrata: Black, non-Black, and Unknown race/ethnicity. At the request of the CCO, all participants of the Health Resilience Program (HRP) who had not been randomly selected into the other strata were added, such that 100% of all HRP participants were included in the sample. HRP participation for these individuals was treated as a fourth substrata, along with the three race categories. In total, there were 16 mutually exclusive sample cells.

Once cases in the sampling universe were placed in one of the sampling cells, sampling targets were set in each cell to create adequate subgroup sizes for the planned analyses, and then a random sample of members from all cases contained within each sampling cell was conducted.

We ended up with a sample of 9,176 cases to survey. Response rates tend to be low in Medicaid surveys, creating the risk of non-response bias, so an intensive follow-up arm was also included. At study onset, 2,000 participants (22% of the sample) was randomly selected to be in the intensive follow-up arm. The survey was fielded in 2015 and 2016. Selected participants received the mail survey with a $5 incentive for participation. The full sample received two additional mailings and telephone reminder calls, and those in the intensive follow-up arm received door-to-door follow-up. A total of 2,348 completed surveys were returned for a response rate of 26%.

Gilmer, T., Kronick, R., Fishman, P., & Ganiats, T. G. (2001). The Medicaid Rx model: Pharmacy-based risk adjustment for public programs. *Medical Care, 39*(11), 1188–1202.

**WEIGHTING**

Weighting was used to allow for an estimate of overall population prevalence that accounted for our sampling design. Within each sampling unit, each case received a weight $W_{i}$: $W_{i}=w_{ij}f_{ik}$, where $w_{ij}$ is the individual’s base weight, calculated as the reciprocal of the probability of inclusion of the individual $i$ in sampling stratum $j$, and $f_{ik}$ is an adjustment factor for individuals in the intensive follow up arm, calculated as the reciprocal of the probability of individual $i$ in the intensive arm $k$ (so that responders to the intensive follow-up arm were able to “stand in” for the larger pool of non-responders in the main study, because they would have been non-responders if the more intensive approach had not existed.).

**SURVEY QUESTIONS**

The purpose of this survey is to learn about your life experiences and how they may have shaped your

health today. This survey will ask you about important events that have happened in your life. We will use

the information from the survey to help build knowledge and help others in the future.

All questions on this survey refer to your health and life experiences. Any personal information you share

will be kept strictly private. When finished, please mail the survey in the included postage-paid envelope.

**Primary Exposures**

*Loss of support*

These next questions ask about the relationships and support you had as an adult.

|  | Early Adulthood  (Ages 19-30) | | Later Adulthood  (Ages 31+) | |
| --- | --- | --- | --- | --- |
|  | Yes | No | Yes | No |
| Did you have close relationships with people you could count on? | ⭘ | ⭘ | ⭘ | ⭘ |

*Loss of financial stability, loss of housing*

These next questions ask about the safety and security you had as an adult. Remember, your answers are

private.

|  | Early Adulthood  (Ages 19-30) | | Later Adulthood  (Ages 31+) | |
| --- | --- | --- | --- | --- |
|  | Yes | No | Yes | No |
| Did you ever have trouble affording enough to eat? | ⭘ | ⭘ | ⭘ | ⭘ |
| Were you ever homeless? | ⭘ | ⭘ | ⭘ | ⭘ |

*Substance use cessation*

These next questions ask about the difficult things that may have happened to you as an adult. Remember,

your answers are private.

|  | Early Adulthood  (Ages 19-30) | | Later Adulthood  (Ages 31+) | |
| --- | --- | --- | --- | --- |
|  | Yes | No | Yes | No |
| Were you a problem drinker, alcoholic, or user of street drugs? | ⭘ | ⭘ | ⭘ | ⭘ |

**Primary Outcome**

*DV perpetration*

These next questions ask about the difficult things that may have happened to you as an adult. Remember,

your answers are private.

|  | Early Adulthood  (Ages 19-30) | | Later Adulthood  (Ages 31+) | |
| --- | --- | --- | --- | --- |
|  | Yes | No | Yes | No |
| Did you slap, hit, kick, punch, or beat up a partner or loved one? | ⭘ | ⭘ | ⭘ | ⭘ |

**Covariates**:

*Sex*

Are you male or female?

⭘ Male ⭘ Female

*Age*

What year were you born? 19___

*Race/ethnicity*

Would you describe yourself as being or Hispanic or Latino/a origin or descent?

⭘ Yes, Hispanic or Latino/a

⭘ No, not Hispanic or Latino/a

How would you describe your race? Mark all that apply.

⭘ White

⭘ Black or African-American

⭘ American Indian

⭘ Asian

⭘ Native Hawaiian or Pacific Islander

⭘ Other ______________________

*Income*:

What was your gross household income (before taxes and deductions are taken out) for last year (2014)? Your best estimate is fine.

⭘ $0

⭘ $1 to $5,000

⭘ $5,001 to $10,000

⭘ $10,001 to $15,000

⭘ $15,001 to $20,000

⭘ $20,001 to $25,000

⭘ $25,001 to $30,000

⭘ $30,001 to $35,000

⭘ $35,001 to $40,000

⭘ $40,001 or more

*Child abuse*

These next questions ask about the difficult things that may have happened to you as a child. Remember,

your answers are private.

|  | Early Childhood (Ages 0-5) | | Early School  (Ages 6-12) | | Teenage Years  (Ages 13-18) | |
| --- | --- | --- | --- | --- | --- | --- |
|  | Yes | No | Yes | No | Yes | No |
| Did an adult in your household swear at you, insult or humiliate you, or act in a way that made you afraid you might be physically hurt? | ⭘ | ⭘ | ⭘ | ⭘ | ⭘ | ⭘ |
| Did an adult ever push, grab, slap, throw something at you, or hit you so hard you had marks or were injured? | ⭘ | ⭘ | ⭘ | ⭘ | ⭘ | ⭘ |
| Did an adult or someone much older than you ever touch or fondle you in a sexual way, OR attempt or actually have sexual intercourse with you? | ⭘ | ⭘ | ⭘ | ⭘ | ⭘ | ⭘ |

*Childhood social support*

These next questions ask about the relationships and support you had as a child. For each question, please

tell us what things were like in your early childhood, early school, and teenage years:

|  | Early Childhood (Ages 0-5) | | Early School  (Ages 6-12) | | Teenage Years  (Ages 13-18) | |
| --- | --- | --- | --- | --- | --- | --- |
|  | Yes | No | Yes | No | Yes | No |
| Did you generally have close, supportive relationships with family and/or friends? | ⭘ | ⭘ | ⭘ | ⭘ | ⭘ | ⭘ |

*Bullying*

These next questions ask about your school years. Tell us about your early school years (ages 6-12) and your

school during your teenage years (13-18). Remember, your answers are private.

|  | Early School  (Ages 6-12) | | Teenage Years  (Ages 13-18) | |
| --- | --- | --- | --- | --- |
|  | Yes | No | Yes | No |
| Were you ever bullied by a peer or classmate? | ⭘ | ⭘ | ⭘ | ⭘ |

*Physical DV victimization*

These next questions ask about the difficult things that may have happened to you as an adult. Remember,

your answers are private.

|  | Early Adulthood  (Ages 19-30) | | Later Adulthood  (Ages 31+) | |
| --- | --- | --- | --- | --- |
|  | Yes | No | Yes | No |
| Were you slapped, hit, kicked, punched, or beat up by a partner or loved one? | ⭘ | ⭘ | ⭘ | ⭘ |
